# Supplementary material for: Transcriptional heterogeneity shapes stress-adaptive responses in yeast
Source: Nat Commun. 2025 Mar 17;16:2631. doi: 10.1038/s41467-025-57911-6 (PMC11914649; doi:10.1038/s41467-025-57911-6)
Supplement: Supplementary file 1 — Supplementary Information [file 41467_2025_57911_MOESM1_ESM.pdf]

Supporting information

Transcriptional heterogeneity shapes stress-adaptive responses in yeast

Mariona Nadal-Ribelles[1,2,*], Guillaume Lieb[3], Carme Sole[1,2], Yaima Matas[1,2], Ugo Szachnowski[4], Sara Andjus[4], Maria Quintana[1,2], Mònica Romo[1,2], Aitor Gonzalez Herrero[1,2], Antonin Morillon[4], Serge Pelet[3], Eulàlia de Nadal[1,2,*], Francesc Posas[1,2,*]

[1]Department of Medicine and Life Sciences, Universitat Pompeu Fabra. Barcelona 08003, Spain

[2]Institute for Research in Biomedicine (IRB Barcelona), the Barcelona Institute of Science and Technology. Barcelona 08028, Spain

[3]Department of Fundamental Microbiology, Faculty of Biology and Medicine, University of Lausanne, Lausanne, Switzerland

[4]ncRNA, Epigenetic and Genome Fluidity, Institut Curie, PSL Research University, Université Pierre et Marie Curie, CNRS UMR 3244, Paris, France

[*]Corresponding authors; mariona.nadal@irbbarcelona.org (MNR); eulalia.nadal@irbbarcelona.org (EdeN), francesc.posas@irbbarcelona.org (FP).

The file includes:

Supplementary Figures 1 to 6

Materials and Methods

Supplementary tables:

Supplementary Table 1. Primers used in this study.

Supplementary Table 2. Yeast strains used in this study.

Supplementary Table 3. Plasmids used in this study.

Methods References

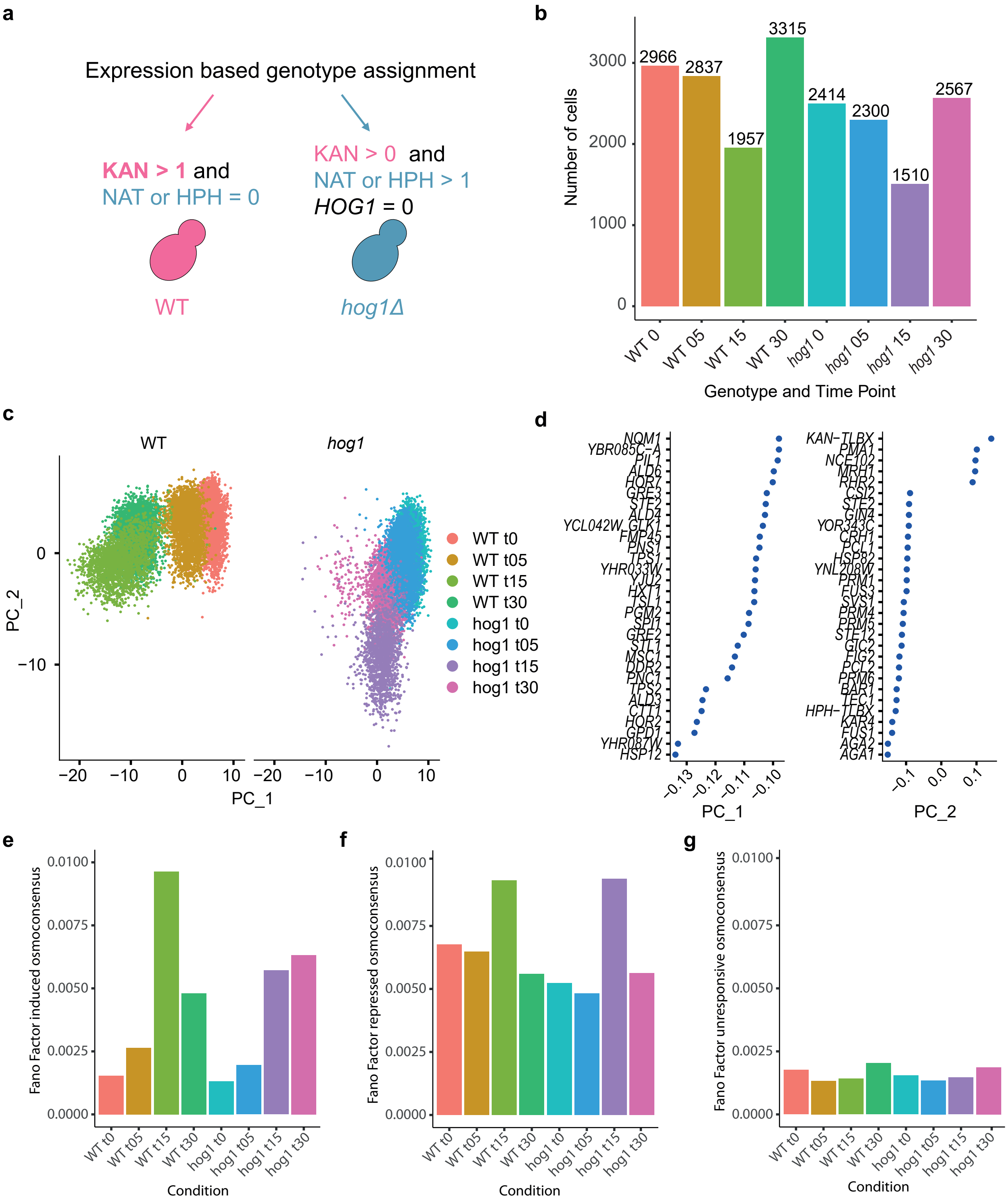
a
Expression based genotype assignment
KAN > 1 and
NAT or HPH = 0
WT
KAN > 0 and
NAT or HPH > 1
HOG1 = 0
hog1Δ
b
Number of cells
2966
2837
1957
3315
2414
2300
1510
2567
WT 0
WT 05
WT 15
WT 30
hog1 0
hog1 05
hog1 15
hog1 30
Genotype and Time Point
c
WT
hog1
PC_2
PC_1
WT t0
WT t05
WT t15
WT t30
hog1 t0
hog1 t05
hog1 t15
hog1 t30
d
NQM1
YBR085C−A
PIL1
ALD6
HOR7
GRE3
STF2
ALD4
YCL042W
GLK1
FMP45
PNS1
TPS1
YHR033W
YJU2
HXT1
PGM2
SPI1
GRE2
SOL1
MSC1
DDR2
PNC1
TPS2
ALD3
CTT1
HOR2
GPD1
YHR087W
HSP12
KAN−TLBX
PMA1
NCE102
MRH1
RHR2
CSI2
STE2
GIN4
YOR343C
CRH1
PCL1
HSP82
YNL208W
PRM1
FUS3
SVS1
PRM4
PRM5
STE12
GIC2
FIG2
PCL2
PRM6
BAR1
TEC1
HPH−TLBX
KAR4
FUS1
AGA2
AGA1
PC_1
PC_2
e
Fano Factor induced osmoconsensus
Condition
f
Fano Factor repressed osmoconsensus
Condition
g
Fano Factor unresponsive osmoconsensus
Condition
WT t0
WT t05
WT t15
WT t30
hog1 t0
hog1 t05
hog1 t15
hog1 t30

Supplementary Figure 1. Osmoadaptation increases transcriptional heterogeneity.

a Schematic representation of the criteria used to assign wild-type and *hog1* mutant cells. **b** Number of high quality (singlets, with genotype assigned cells) that passed QC. **c** Principal component clustering of the entire dataset, all timepoints are shown grouped by strain. **d** Visualization of gene loading for PC1 and PC2 genes from (c). Gene names are in the y-axis and PC1 loading values are in the x-axis for the indicated PC dimension.**e-g**) Barplot shows Fano factors (y axis) for the induced (e), repressed (f) and unresponsive osmoconsensus (g) signatures across all samples. Source data are provided as a Source Data file.

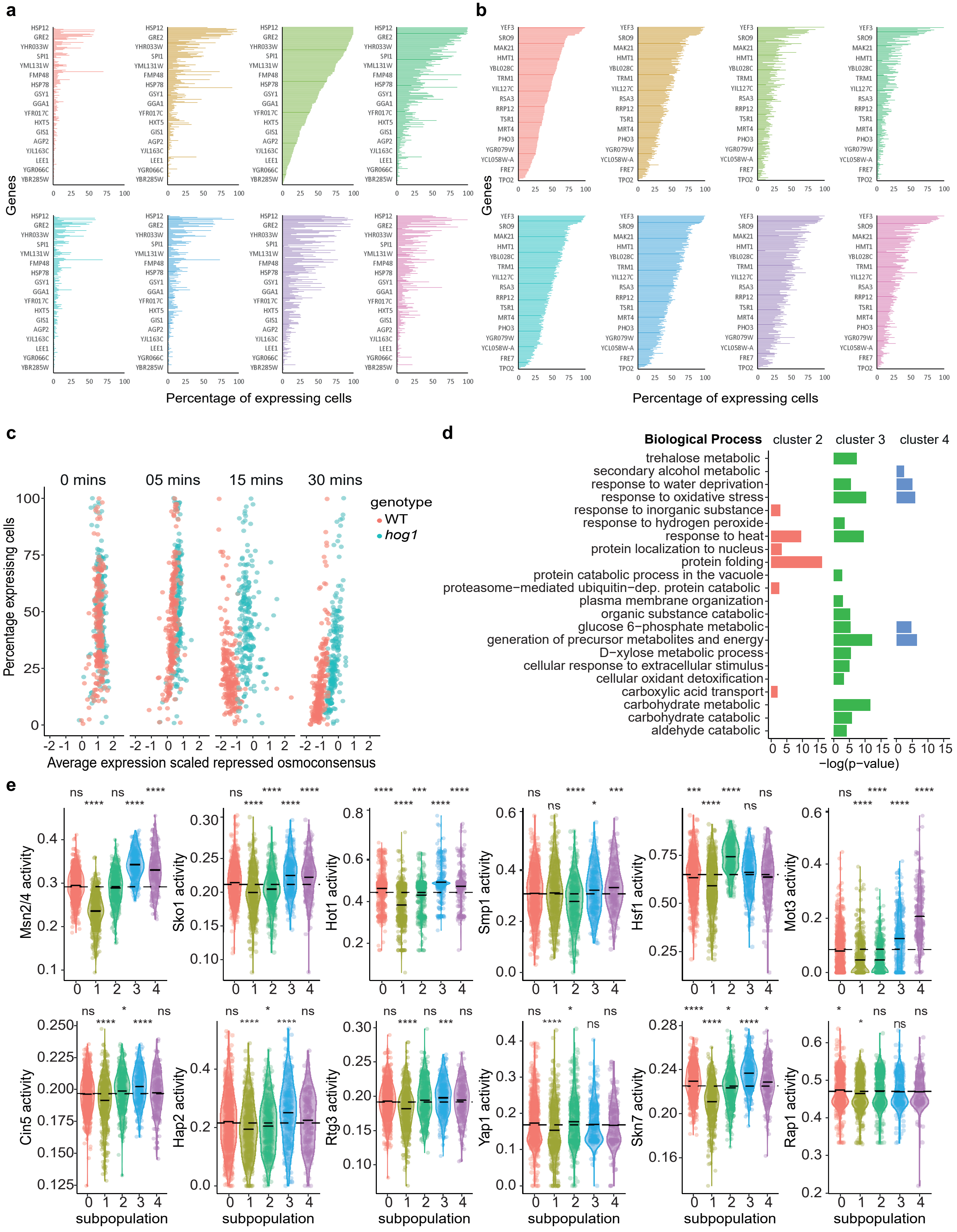

a
b
Genes
Percentage of expressing cells
c
0 mins
05 mins
15 mins
30 mins
genotype
WT
hog1
Percentage expresing cells
Average expression scaled repressed osmoconsensus
d
Biological Process
cluster 2
cluster 3
cluster 4
trehalose metabolic
secondary alcohol metabolic
response to water deprivation
response to oxidative stress
response to inorganic substance
response to hydrogen peroxide
response to heat
protein localization to nucleus
protein folding
protein catabolic process in the vacuole
proteasome−mediated ubiquitin−dep. protein catabolic
plasma membrane organization
organic substance catabolic
glucose 6−phosphate metabolic
generation of precursor metabolites and energy
D−xylose metabolic process
cellular response to extracellular stimulus
cellular oxidant detoxification
carboxylic acid transport
carbohydrate metabolic
carbohydrate catabolic
aldehyde catabolic
−log(p−value)
e
Msn2/4 activity
Sko1 activity
Hot1 activity
Smp1 activity
Hsf1 activity
Mot3 activity
Cin5 activity
Hap2 activity
Rtg3 activity
Yap1 activity
Skn7 activity
Rap1 activity
subpopulation

Supplementary Figure 2. Cells display a heterogeneous use of the osmoresponsive program upon stress.

a-b Global overview of percentage of gene usage for the induced (a) and repressed osmoconsensus program (b). Bar plot indicates the gene names (y-axis) and the percentage of expressing cells (x-axis) for the indicated strain and time. In (a), all panels display genes listed in descending order using the wild type treated with NaCl 0.4M, 15 min dataset as a reference. In (b), genes are listed in descending order using the wild type strain in control conditions. **c** Scatter plot represents the scaled average expression of each repressed osmoconsensus gene (n=200 genes, x-axis) in wild-type (red points) or *hog1* mutant cells (blue points) against the percentage of expressing cells (y-axis) for the indicated times. **d** Gene Ontology enrichment of upregulated marker genes for cluster 2 (red bars) and 3 (green bars) from (2G). **e** Violin plot represents the expression distribution of the transcription factor annotated target genes from each cluster obtained from the Saccharomyces Gene Database (SGD). Black thick lines represent the median of each subpopulation whereas the dashed black line represents the population median. Two sided Wilcoxon test with Benjamini-Hochberg adjustment is shown. Source data are provided as a Source Data file.

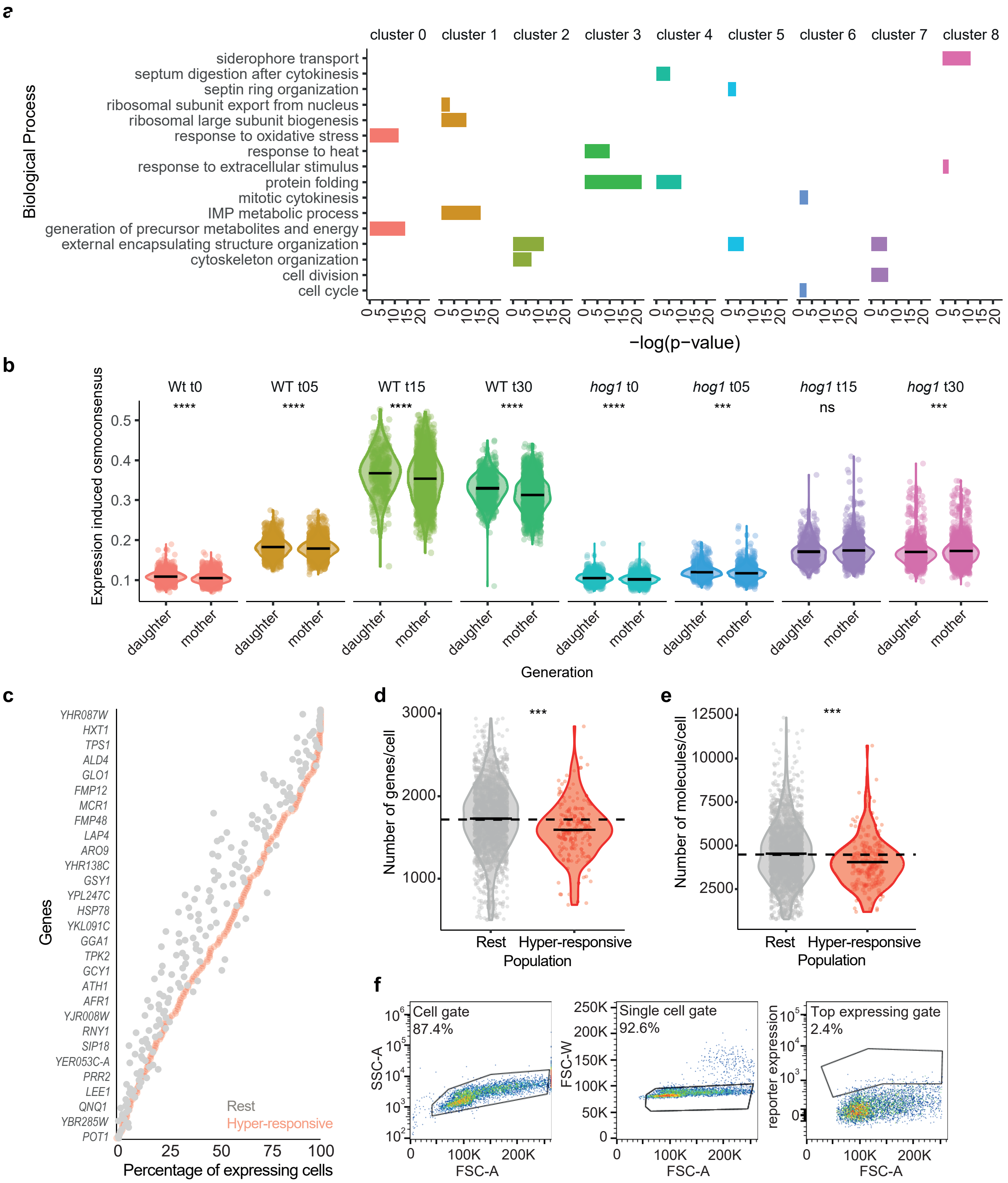
a
cluster 0
cluster 1
cluster 2
cluster 3
cluster 4
cluster 5
cluster 6
cluster 7
cluster 8
Biological Process
siderophore transport
septum digestion after cytokinesis
septin ring organization
ribosomal subunit export from nucleus
ribosomal large subunit biogenesis
response to oxidative stress
response to heat
response to extracellular stimulus
protein folding
mitotic cytokinesis
IMP metabolic process
generation of precursor metabolites and energy
external encapsulating structure organization
cytoskeleton organization
cell division
cell cycle
−log(p−value)
b
Expression induced osmoconsensus
Wt t0
WT t05
WT t15
WT t30
hog1 t0
hog1 t05
hog1 t15
hog1 t30
****
***
ns
daughter
mother
Generation
c
Genes
YHR087W
HXT1
TPS1
ALD4
GLO1
FMP12
MCR1
FMP48
LAP4
ARO9
YHR138C
GSY1
YPL247C
HSP78
YKL091C
GGA1
TPK2
GCY1
ATH1
AFR1
YJR008W
RNY1
SIP18
YER053C-A
PRR2
LEE1
QNQ1
YBR285W
POT1
Rest
Hyper-responsive
Percentage of expressing cells
d
Number of genes/cell
Rest
Hyper-responsive
Population
e
Number of molecules/cell
Rest
Hyper-responsive
Population
f
Cell gate
87.4%
SSC-A
FSC-A
Single cell gate
92.6%
FSC-W
Top expressing gate
2.4%
reporter expression

Supplementary Figure 3. Transcriptional heterogeneity shapes the adaptive capacity of the cells.

a Gene Ontology enrichment using the upregulated genes for each cluster in the wild type 15 min 0.4M NaCl dataset. Plot shows the top 2 Biological Process for the indicated clusters. **b** Expression distribution of induced osmoconsensus program for the indicated times and strains. Cells were classified into daughters based on the expression signature of known daughter gene signature; the rest of the cells were considered mothers. Statistical significance of the comparison between daughters and mothers for each comparison is shown (Wilcoxon test). **c** Percentage of expression of the induced osmoconsensus program in cells considered hyper-responsive (red dots) and the rest of the population (grey dots). Gene names are shown in the y axis and a representative gene (*HXT5*) selected for experimental validation is highlighted. **d-e** Distribution of the filtered raw number of genes (d) and number of molecules (e) for the hyper-responsive (red) and the rest of the population (grey). Points represent cells in the indicated populations. Black line represents the mean abundance while dotter lines the population mean. Statistical significance (Two sided Wilcoxon test Benjamini-Hochberg adjusted) is shown between groups. **f.** Cells were gated sequentially using first the FSC-A/SSC-A to define the cell gate, then FSC-A/FSC-W to define single cell particles, and reporter fluorescence (mCherry-FSC-A) to isolate the top 2% expressing populations. For competition assays and expression analysis, cells were gated using FSC-A/SSC-A and analyzed for gene expression or population of interest. Source data are provided as a Source Data file.

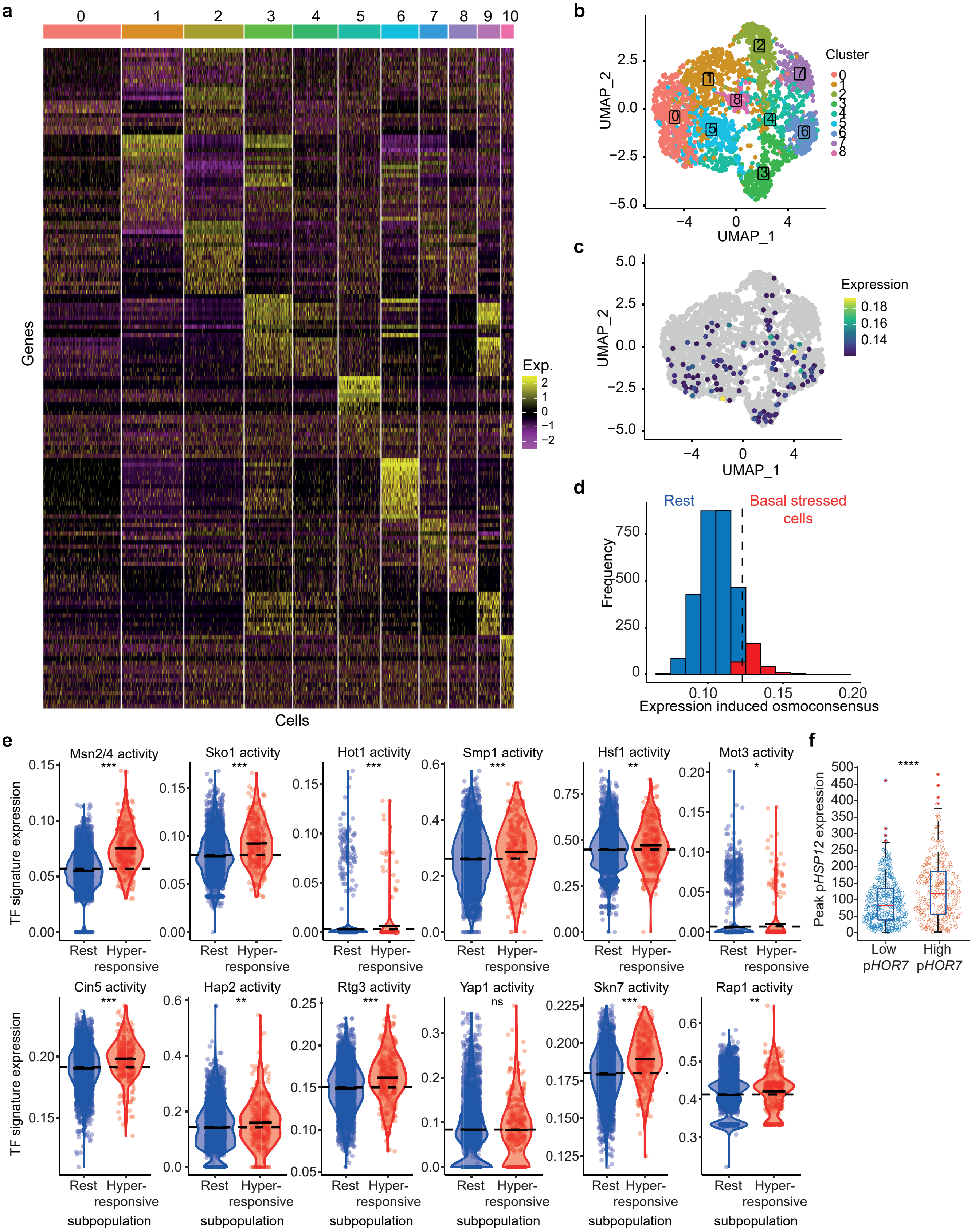

a
Genes
Cells
Exp.
b
UMAP_1
UMAP_2
Cluster
c
Expression
d
Rest
Basal stressed cells
Frequency
Expression induced osmoconsensus
e
Msn2/4 activity
Sko1 activity
Hot1 activity
Smp1 activity
Hsf1 activity
Mot3 activity
Cin5 activity
Hap2 activity
Rtg3 activity
Yap1 activity
Skn7 activity
Rap1 activity
TF signature expression
Rest
Hyper-responsive
subpopulation
f
Peak pHSP12 expression
Low pHOR7
High pHOR7

Supplementary Figure 4. Stochastic expression of stress programs in the absence of stress.

a Heatmap represents the top 20 marker genes for each cluster for the wild-type control condition. Warmer colors indicate higher expression. **b** Louvain clustering and projection onto wild-type control UMAP, cluster number is shown on top for *hog1* mutant cells in control. **c** UMAP projection of cells basally stressed based on the expression of the induced osmoconsensus signature. Highlighted cells are above the minimum cut off and colored based on the expression level for *hog1* mutant cells in the control. Warmer colors indicate higher expression of the signature. **d** Expression distribution of wild-type control cells. Vertical line represents the threshold used for the classification of basal stress-primed cells (orange) or the rest of the population (blue). **e** Expression distribution of the basal stressed population (blue) and the remaining cells (orange) for the indicated transcription factor signatures. Each signature represents the transcription factor-regulated genes extracted from the Saccharomyces Gene Database (SGD). Wilcoxon test is shown above each transcription factor comparison. **f** Comparison of the p*HSP12*-PP7-mCherry reporter from Figure 4e. Single cell traces were sorted in two sub-populations based on the level p*HOR7*-MS2-GFP under basal conditions. For each cell in each subpopulation the maximal *HSP12* expression is plotted (points). The expression distribution of each subpopulation is shown (boxplot) and the two sided t-test was performed to compare the mean expression between the low and high cells (p-value). Box plot represent the median (center), 25th and 75th percentile (box) and whiskers represent the minima and maxima not considering outliers (1.5x the difference between the 25th and the 75th percentiles. The box spans from the 25th percentile (Q1) to the 75th percentile (Q3), with the central line indicating the median. The whiskers extend to the most extreme data points within 1.5 times the interquartile range (Q3-Q1) over and below Q1 and Q3. Data points outside this range are considered outliers and plotted with red crosses. Symbols ns/*/**/***/**** represent p-values >0.05, <0.05, <0.01, <0.001, <0.0001. Source data are provided as a Source Data file.

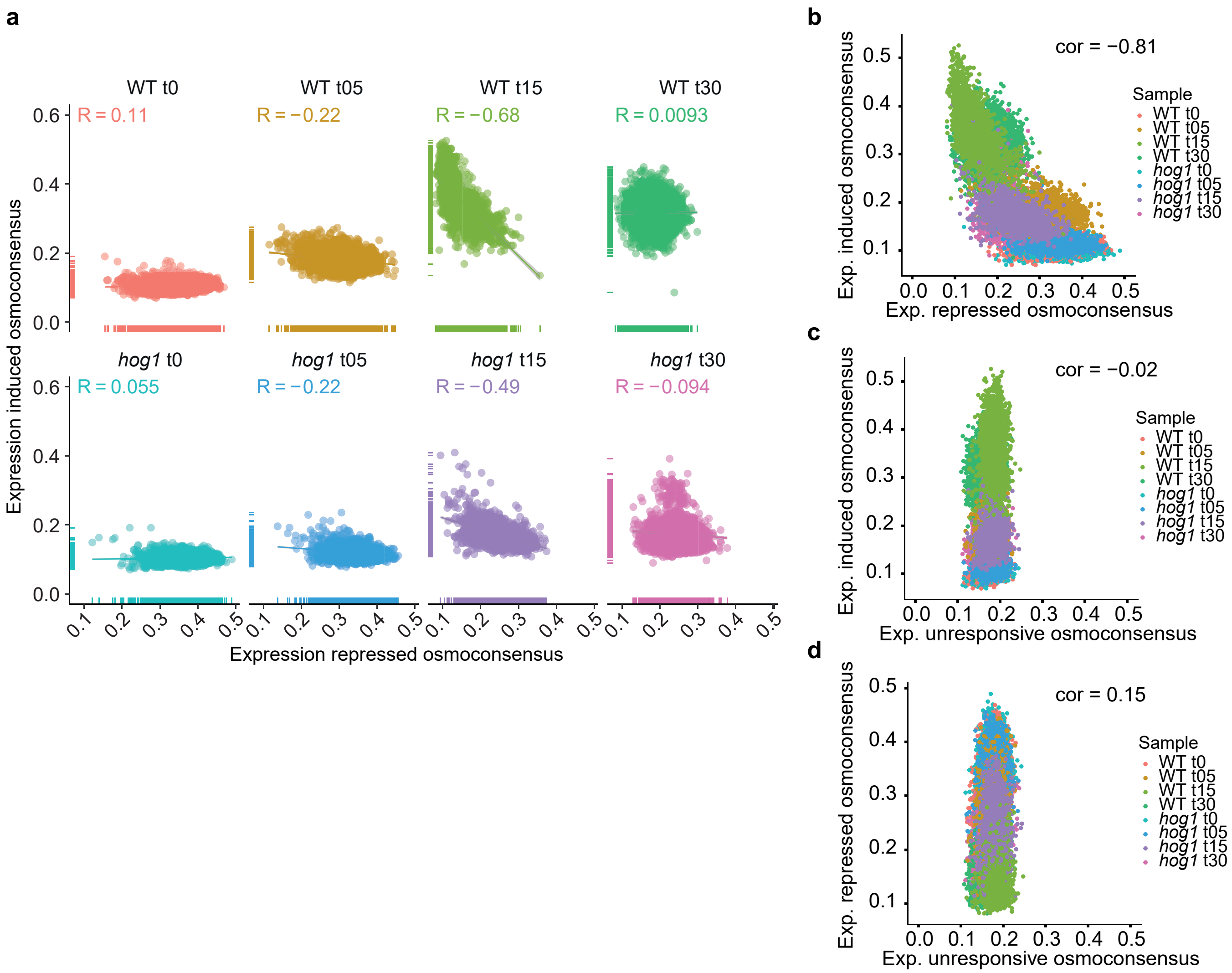
a
WT t0
WT t05
WT t15
WT t30
R = 0.11
R = −0.22
R = −0.68
R = 0.0093
hog1 t0
hog1 t05
hog1 t15
hog1 t30
R = 0.055
R = −0.22
R = −0.49
R = −0.094
Expression induced osmoconsensus
Expression repressed osmoconsensus
b
cor = −0.81
Exp. induced osmoconsensus
Exp. repressed osmoconsensus
Sample
WT t0
WT t05
WT t15
WT t30
hog1 t0
hog1 t05
hog1 t15
hog1 t30
c
cor = −0.02
Exp. induced osmoconsensus
Exp. unresponsive osmoconsensus
d
cor = 0.15
Exp. repressed osmoconsensus
Exp. unresponsive osmoconsensus

Supplementary Figure 5. Global transcriptome repression favors induction of the osmoresponsive program.

a Per cell expression correlation of the induced osmoconsensus score (y-axis) against the score of repressed genes (x-axis) for the indicated strains and conditions. Points are colored according to their condition and a marginal lines in both axes represent the distribution of data points. Linear regression with 0.95 confidence interval is shown and the Pearson correlation coefficient (r) for each facet is shown above the plot area. **b-d** Expression correlation for the indicated signatures. Cells are colored according to the condition, and Pearson correlation value is shown. Source data are provided as a Source Data file.

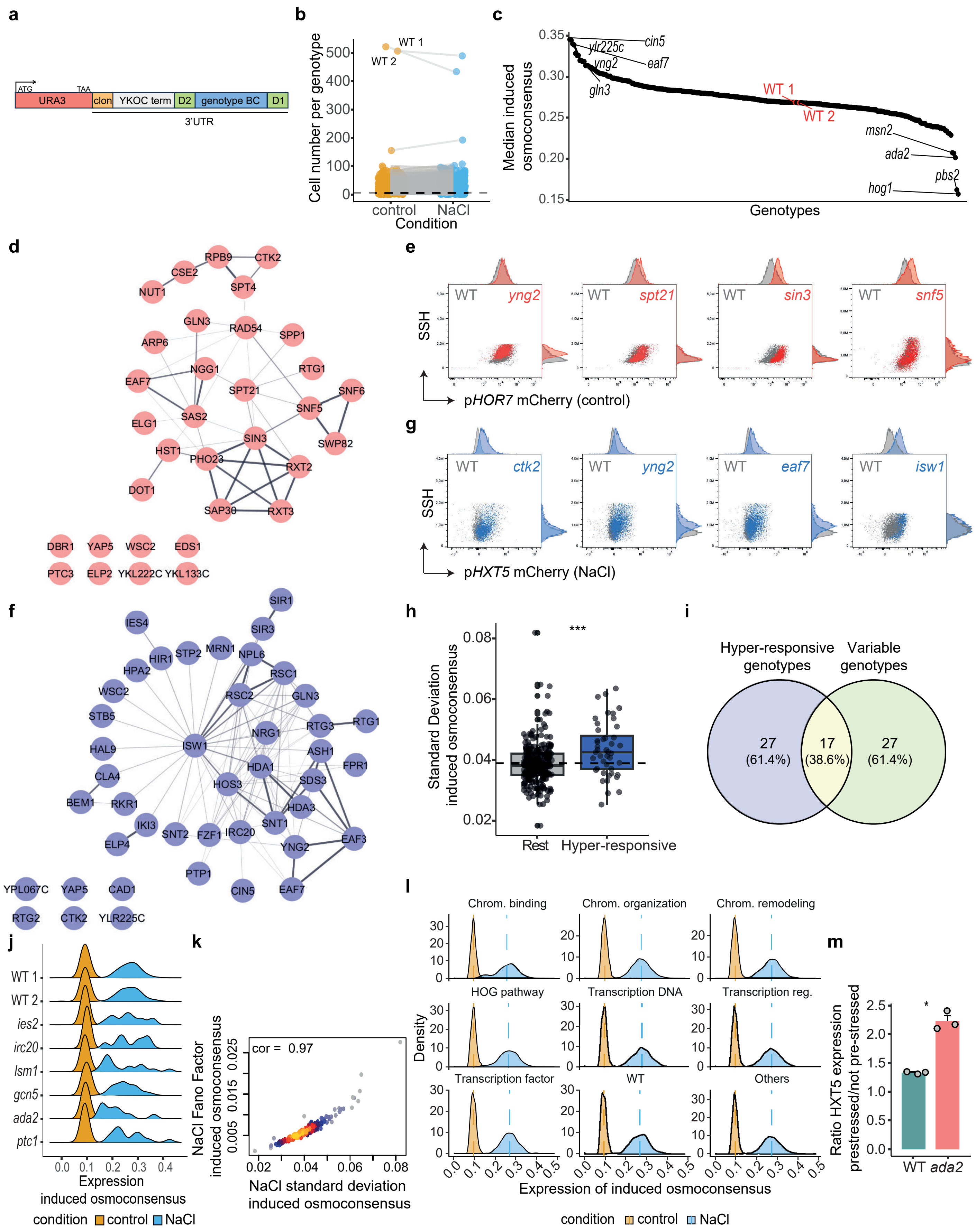
a
b
c
ATG
TAA
URA3
clon
YKOC term
D2
genotype BC
D1
3'UTR
Cell number per genotype
WT 1
WT 2
control
NaCl
Condition
Median induced osmoconsensus
cin5
ylr225c
eaf7
yng2
gln3
WT 1
WT 2
msn2
ada2
pbs2
hog1
Genotypes
d
RPB9
CTK2
CSE2
NUT1
SPT4
GLN3
RAD54
SPP1
ARP6
NGG1
RTG1
EAF7
SPT21
SNF6
SNF5
ELG1
SAS2
SIN3
SWP82
HST1
PHO23
RXT2
DOT1
SAP30
RXT3
DBR1
YAP5
WSC2
EDS1
PTC3
ELP2
YKL222C
YKL133C
e
WT
yng2
spt21
sin3
snf5
SSH
pHOR7 mCherry (control)
g
ctk2
eaf7
isw1
pHXT5 mCherry (NaCl)
f
SIR1
IES4
SIR3
MRN1
HIR1
STP2
NPL6
HPA2
RSC1
WSC2
RSC2
GLN3
STB5
NRG1
RTG3
RTG1
HAL9
ISW1
ASH1
HDA1
FPR1
CLA4
HOS3
SDS3
BEM1
RKR1
HDA3
IKI3
SNT1
SNT2
FZF1
IRC20
ELP4
YNG2
EAF3
PTP1
CIN5
EAF7
YPL067C
CAD1
RTG2
YLR225C
h
Standard Deviation induced osmoconsensus
***
Rest
Hyper-responsive
i
Hyper-responsive genotypes
Variable genotypes
27 (61.4%)
17 (38.6%)
27 (61.4%)
j
WT 1
WT 2
ies2
irc20
lsm1
gcn5
ada2
ptc1
Expression induced osmoconsensus
condition
control
NaCl
k
NaCl Fano Factor induced osmoconsensus
cor = 0.97
NaCl standard deviation induced osmoconsensus
l
Chrom. binding
Chrom. organization
Chrom. remodeling
HOG pathway
Transcription DNA
Transcription reg.
Transcription factor
WT
Others
Density
Expression of induced osmoconsensus
condition
control
NaCl
m
Ratio HXT5 expression prestressed/not pre-stressed
*
WT
ada2

Supplementary Figure 6. A genetic screen served to identify key elements of the osmoadaptive transcriptional phenotypes.

a Schematic representation to generate RNA-barcoded mutants. **b** Plot shows the total number of cells assigned to each genotype in control and stress conditions after QC and removing unassigned genotypes. **c** Expression distribution of the induced osmoconsensus signature across each genotype (x-axis). Names of top and bottom genotypes are displayed. **d** STRING network of mutants with high basal expression of the induced osmoconsensus signature (red points from Figure 6f). Nodes are connected by experimental evidence, and lines represent confidence (minimum 0.150). **e** Representative scatter plot of pHOR7-UbiM-mCherry-tHOR7 expression against side scatter (SSH) by FACS for basal stressed mutants in control conditions. Marginal histograms are shown. **f** Physical protein-protein interaction networks as in (d) of mutants that, in stress conditions, have a high percentage of hyper-responsive cells (blue points from Figure 6h). **g** Scatter plot of p*HXT5*-UbiM-mCherry-t*HXT5* expression (x-axis) against SSH (y-axis) for hyper-responsive mutants under stress (0.4M NaCl, 1h). Histograms are shown next to each axis. **h** Distribution of the standard deviation of the induced osmoconsensus signature for mutants containing a higher fraction of osmoresponsive cells (blue points from Figure 6h). Dotted line indicates the population. Two-sided Wilcoxon test is shown (p-value 0.00064). **i** Overlap of hyper-responsive mutants (Figure 6h) and highly heterogeneous mutants from Figure 6j. **j** Density plots show the single-cell expression distribution of the induced osmoconsensus signature of mutants and the wild-type in control (yellow) or stress (blue) histograms. **k** Scatter plot comparing the standard deviation and Fano factor are calculated for the induced osmoconsensus upon stress. Points are colored by density, with yellow indicating the highest point density. Spearman correlation is shown. **l** Histograms show the standard deviation of the induced osmoconsensus signature in control (red histogram) and stress (blue histograms) conditions. Each plot contains the mutants associated with each GO category (expression mean is shown as a dotted line) **m** Expression of destabilized *HXT5* reporter in the indicated strains. Cells were pre-stressed or not in the presence of mild osmostress (0.4M NaCl 30 mins) and then subjected to hyperosmotic stress to the same final NaCl concentration (1M). For each strain the median expression of *HXT5* mCherry signal was extracted (n=3). Two sided Wilcoxon test is shown. Source data are provided as a Source Data file.

Supplementary Table 1. Primers used in this study.

Number Oligo	Gene	Name Oligo	Sequence	Source
OMN852	C-term tag *TDH3*	*TDH3*_s2_Fw	ATACGGTTACTCTACCAGAGTTGTCGACTTGGTTGAA CACGTTGCCAAGGCT CGTACGCTGCAGGTCGAC	This study
OMN851	C-term tag *TDH3*	*TDH3*_s2_Rv	gctataaaaagaaaatttatttaaatgcaagatttaaagtaaattcac TTA ATCGATGAATTCGAGCTCG	This study
OMN966	mCherry 3b	Fw_mCherry _MoClo_3b	GCATCGTCTCATCGGTCTCATTCTATGGTGAGCAAGG GCGAGGA	This study
OMN967	mCherry 3b	Rv_mCherry_ MoClo_3b	ATGCCGTCTCAGGTCTCAGGATCCCTACTTGTACAGC TCGTCCATG	This study
ORP106	*HOR7*	Fw_p*HOR7*_ MoClo2	GCATCGTCTCATCGGTCTCAAACGTCACATAGACTGC GTCATAAAAATAC	This study
ORP107	*HOR7*	Rv_p*HOR7*_ MoClo2	ATGCCGTCTCAGGTCTCACATATTTTTATTATTAGTCT TTTTTTTTTTTG	This study
ORP108	*HOR7*	Fw_t*HOR7*_ MoClo4	GCATCGTCTCATCGGTCTCAATCCTAACTCGAGATTTA GACTTTGTTTGGAATACATTGA	This study
ORP109	*HOR7*	Rv_t*HOR7*_M oClo4	ATGCCGTCTCAGGTCTCACAGCCTTATTTTTGGGTTCT TTAGAGGAAAAAATTTTCGATT	This study
OMN960	*HXT5*	Fw_p*HXT5*_ MoClo_2	GCATCGTCTCATCGGTCTCAAACG ACTAAAAAGGTGGCCCATCC	This study
OMN961	*HXT5*	Rv_p*HXT5*_M oClo_2	ATGCCGTCTCAGGTCTCACATATCTTTCTTTACCTCTA ATATATTC	This study
OMN964	*HXT5*	Fw_t*HXT5*_M oClo_4	GCATCGTCTCATCGGTCTCAATCCTAACTCGAG TCTCTTGTAGTGTAGGATCAAC	This study
OMN965	*HXT5*	Rv_t*HXT5*_M oClo_4	ATGCCGTCTCAGGTCTCACAGCTAAGTAACAAGAAAC GATATACAAG	This study

Supplementary Table 2. Strains used in this study.

Strain name	Genotype	Source
YMN478	BY4741 *TEF1*-URA3 (genotype barcode, cgcacgaatatttttcttag) wild type for deletion based scRNA-seq	Nadal-Ribelles et al. [18]
YMN479	BY4741 *TEF1*-URA3 (genotype barcode, agatcgtagaccgcatctac) wild type for deletion based scRNA-seq	Nadal-Ribelles et al. [18]
yRP289	BY4741-pYTK778-*pHXT5*-UbiM-mCherry 3b-*tHXT5:HIS*	This study
yRP298	YMN479 -pYTK778-*pHXT5-UbiM*-mCherry 3b-*tHXT5:HIS*	This study
yRP304	*YJL006C*::URA3-pYTK778-*pHXT5*-UbiM-mCherry-3b-*tHXT5:HIS*	This study
yRP317	*YHR090C*::URA3-pYTK778-*pHXT5*-UbiM-mCherry-3b-*tHXT5:HIS*	This study
yRP332	*YNL136W*::URA3-pYTK778-*pHXT5*-UbiM-mCherry-3b-*tHXT5:HIS*	This study
yRP384	*YBR245C*::URA3-pYTK778-pHXT5-UbiM-mCherry-3b-tHXT5:HIS	This study
yRP350	BY4741-pYTK168-*pHOR7*-UbiM-mCherry 3b-*tHOR7:NAT*	This study
yRP359	YMN479-pYTK168-*pHOR7*-UbiM-mCherry 3b-*tHOR7:NAT*	This study
yRP465	BY4741-pYTK147-pSAG1-UbiM-mCherry 3b- tSAG1:HIS	This study
yRP362	*YHR090C*::URA3-pYTK168-*pHOR7*-UbiM-mCherry-3b-*tHOR7:NAT*	This study
yRP365	*YMR179W*::URA3-pYTK168-p*HOR7*-UbiM-mCherry-3b-*tHOR7:NAT*	This study
yRP368	*YOL004W*::URA3-pYTK168-*pHOR7*-UbiM-mCherry-3b-*tHOR7:NAT*	This study
yRP417	*YBR289W::URA3-pYTK168-pHOR7-UbiM-mCherry 3b-tHOR7:NAT*	This study
yRP350	BY4741-pYTK168-*pHOR7*-UbiM-mCherry 3b-*tHOR7:NAT* (Clone 1)	This study
yGL42	HTA2-tdiRFP:TRP/ HTA2-tdiRFP:NAT; GLT1::pHIS-pHSP12 PP7sl/ GLT1:: pHIS-pEFT2 24xMSsl; URA3::pSIVura pADH-PP7-mCherry URA3::pSIVura pADH-PP7-GFPenvy	This study
yGL43	HTA2-tdiRFP:TRP/ HTA2-tdiRFP:NAT; GLT1::pHIS-pHSP12 PP7sl/ GLT1:: pHIS-pHXT5 24xMSsl; URA3::pSIVura pADH-PP7-mCherry URA3::pSIVura pADH-PP7-GFPenvy	This study
yGL46	HTA2-tdiRFP:TRP/ HTA2-tdiRFP:NAT; GLT1::pHIS-pHSP12 PP7sl/ GLT1:: pHIS-pHOR7 24xMSsl; URA3::pSIVura pADH-PP7-mCherry URA3::pSIVura pADH-PP7-GFPenvy	This study
YMN_Z03E03	*YJL124C::URA3*	This study
YMN_Z04F09	*YDR448W::URA3*	This study
YMN_Z01G01	*YDL006W::URA3*	This study
YMN_Z01B07	*YBR200W::URA3*	This study
YMN_Z04D06	*YNL215W::URA3*	This study
YMN_Z04F02	*YBL103C::URA3*	This study
YMN_Z01D05	*YPR179C::URA3*	This study
YMN_Z01F06	*YNL023C::URA3*	This study
YMN_Z01D08	*YPR189W::URA3*	This study
YMN_Z02E05	*YMR091C::URA3*	This study
YMN_Z01C12	*YKL149C::URA3*	This study
YMN_Z01B01	*YNL298W::URA3*	This study
YMN_Z02C12	*YKL043W::URA3*	This study

Supplementary Table 3. Plasmids used in this study.

Plasmid number	Plasmid Name	Description	Source
pDC644	pMYT079-HIS3 (pYTK778)	backbone for integrative plasmids	This study
pNO82	pYTK-Nat-HO_int-Nat (pYTK168)	backbone for integrative plasmids	Canadell et al. [19]
pRP144	pYTK778-pHXT5-UbiM-mCherry 3b-tHXT5	Integration plasmid carrying the promoter of *HXT5* an N-terminal degradation signal fused to mCherry and terminator *HXT5*.	This study
pRP153	pYTK168-p*HOR7*-UbiM-mCherry 3b-t*HOR7*	Integration plasmid carrying promoter of HOR7 an N-terminal degradation signal fused to mCherry and terminator HOR7.	This study
pVW293	pHIS-p*HSP12* 24xPP7sl	Integration plasmid carrying the *HSP12* promoter followed by the 24 PP7 stem loops for integration in the *GLT1* locus	Wosika et al 2020[20]
pGL1	pHIS-p*HSP12* 24MS2sl	Integration plasmid carrying the *HSP12* promoter followed by the 24 MS2 stem loops for integration in the *GLT1* locus	This study
pSP680	pHIS-p*EFT2* 24xMS2sl	Integration plasmid carrying the *EFT2* promoter followed by the 24 MS2 stem loops for integration in the *GLT1* locus	This study
pSP682	pHIS-p*HXT5* 24xMS2sl	Integration plasmid carrying the *HXT5* promoter followed by the 24 MS2 stem loops for integration in the *GLT1* locus	This study
pSP680	pHIS-p*HOR7* 24xMS2sl	Integration plasmid carrying the HOR7 promoter followed by the 24 MS2 stem loops for integration in the *GLT1* locus	This study
pVW296	pSIVu p*ADH1*-PP7 mCherry	Integration plasmid expressing the PP7-mCherry construct	Wosika et al 2020[20]
pSP561	pSIVu p*ADH1*-MS2 GFPenvy	Integration plasmid expressing the MS2-GFP construct	Wosika et al 2020[20]
pRP102	pYTK147-p*SAG1*-UbiM-mCherry 3b-t*SAG1*	Integrating plasmid carrying the *SAG1* reporter consisting of the promoter of *SAG1*, an N-terminal degradation signal fused to mCherry and terminator *SAG1*.	This study

Supplementary References

1. Janke, C. *et al.* A versatile toolbox for PCR-based tagging of yeast genes: New fluorescent proteins, more markers and promoter substitution cassettes. *Yeast* **21**, 947–962 (2004).

2. Lee, M. E., DeLoache, W. C., Cervantes, B. & Dueber, J. E. A Highly Characterized Yeast Toolkit for Modular, Multipart Assembly. *ACS Synth Biol* **4**, 975–986 (2015).

3. Pelet, S., Dechant, R., Lee, S. S., Van Drogen, F. & Peter, M. An integrated image analysis platform to quantify signal transduction in single cells. *Integr Biol (Camb)* **4**, 1274–1282 (2012).

4. Pachitariu, M. & Stringer, C. Cellpose 2.0: how to train your own model. *Nature Methods 2022 19:12* **19**, 1634–1641 (2022).

5. Stringer, C., Wang, T., Michaelos, M. & Pachitariu, M. Cellpose: a generalist algorithm for cellular segmentation. *Nature Methods 2020 18:1* **18**, 100–106 (2020).

6. Zheng, G. X. Y. *et al.* Massively parallel digital transcriptional profiling of single cells. *Nat Commun* **8**, 1–12 (2017).

7. Nadal-Ribelles, M. *et al.* Control of Cdc28 CDK1 by a Stress-Induced lncRNA. *Mol Cell* **53**, 549–561 (2014).

8. Satija, R., Farrell, J. A., Gennert, D., Schier, A. F. & Regev, A. Spatial reconstruction of single-cell gene expression data. *Nature Biotechnology 2015 33:5* **33**, 495–502 (2015).

9. Jackson, C. A., Castro, D. M., Saldi, G. A., Bonneau, R. & Gresham, D. Gene regulatory network reconstruction using single-cell rna sequencing of barcoded genotypes in diverse environments. *Elife* **9**, (2020).

10. Zhou, Y. *et al.* Metascape provides a biologist-oriented resource for the analysis of systems-level datasets. *Nature Communications 2019 10:1* **10**, 1–10 (2019).

11. Andreatta, M. & Carmona, S. J. UCell: robust and scalable single-cell gene signature scoring. *Comput Struct Biotechnol J* **19**, 3976–3978 (2021).

12. Latorre, P. *et al.* Data-driven identification of inherent features of eukaryotic stress-responsive genes. *NAR Genom Bioinform* **4**, (2022).

13. Colman-Lerner, A., Chin, T. E. & Brent, R. Yeast Cbk1 and Mob2 Activate Daughter-Specific Genetic Programs to Induce Asymmetric Cell Fates. *Cell* **107**, 739–750 (2001).

14. Thomas, V. N., Weiss, E. L. & Brace, J. L. Asymmetric secretion in budding yeast reinforces daughter cell identity. doi:10.1101/483388.

15. Marsh SE. scCustomize: Custom Visualizations & Functions for Streamlined Analyses of Single Cell Sequencing. Preprint at (2021).

16. Szklarczyk, D. *et al.* The STRING database in 2023: protein-protein association networks and functional enrichment analyses for any sequenced genome of interest. *Nucleic Acids Res* **51**, D638–D646 (2023).

17. Shannon, P. *et al.* Cytoscape: A Software Environment for Integrated Models of Biomolecular Interaction Networks. *Genome Res* **13**, 2498–2504 (2003).

18. Nadal-Ribelles, M. *et al.* Perturbation-driven transcriptional heterogeneity impacts cell fitness. *bioRxiv* 2024.05.31.596868 (2024) doi:10.1101/2024.05.31.596868.

19. Canadell, D. *et al.* Implementing re-configurable biological computation with distributed multicellular consortia. *Nucleic Acids Res* **1**, 1–18 (2022).

20. Wosika, V. & Pelet, S. Single-particle imaging of stress-promoters induction reveals the interplay between MAPK signaling, chromatin and transcription factors. *Nature Communications 2020 11:1* **11**, 1–13 (2020).
