# Supplementary material for: Transcriptional heterogeneity shapes stress-adaptive responses in yeast
Source: Nat Commun. 2025 Mar 17;16:2631. doi: 10.1038/s41467-025-57911-6 (PMC11914649; doi:10.1038/s41467-025-57911-6)
Supplement: Supplementary file 2 — Description of Additional Supplementary Information [file 41467_2025_57911_MOESM2_ESM.pdf]

Description of Additional Supplementary Files:

File Name: Supplementary Data 1
Description: Gene lists used to generate gene expression signatures across all datasets.
File Name: Supplementary Data 2
Description: Percentage gene use osmoconsensus signature across all datasets.
File Name: Supplementary Data 3
Description: Cluster marker genes and Gene Ontology enrichment for WT 15 mins dataset clustered by stress responsive genes.
File Name: Supplementary Data 4
Description: Cluster marker genes and Gene Ontology enrichment for WT 15 mins dataset.
File Name: Supplementary Data 5
Description: Cluster marker genes and Gene Ontology enrichment for WT control dataset.
File Name: Supplementary Data 6
Description: Differential expression table of basal stressed cells.
File Name: Supplementary Data 7
Description: Genotype summary metrics of the induced osmoconsensus signature for the transcription-focused gene deletion dataset including Fano factor.
