# Supplementary material for: Transcriptional heterogeneity shapes stress-adaptive responses in yeast
Source: Nat Commun. 2025 Mar 17;16:2631. doi: 10.1038/s41467-025-57911-6 (PMC11914649; doi:10.1038/s41467-025-57911-6)
Supplement: Supplementary file 10 — Reporting Summary [file 41467_2025_57911_MOESM10_ESM.pdf]

nature portfolio

Corresponding author(s): Francesc Posas
Eulàlia de Nadal
Mariona Nadal-Ribelles

Last updated by author(s): Feb 19, 2025

# Reporting Summary

Nature Portfolio wishes to improve the reproducibility of the work that we publish. This form provides structure for consistency and transparency in reporting. For further information on Nature Portfolio policies, see our Editorial Policies and the Editorial Policy Checklist.

## Statistics

For all statistical analyses, confirm that the following items are present in the figure legend, table legend, main text, or Methods section.

| n/a | Confirmed | |
|---|---|---|
| ☐ | ☒ | The exact sample size (*n*) for each experimental group/condition, given as a discrete number and unit of measurement |
| ☐ | ☒ | A statement on whether measurements were taken from distinct samples or whether the same sample was measured repeatedly |
| ☐ | ☒ | The statistical test(s) used AND whether they are one- or two-sided<br>*Only common tests should be described solely by name; describe more complex techniques in the Methods section.* |
| ☒ | ☐ | A description of all covariates tested |
| ☐ | ☒ | A description of any assumptions or corrections, such as tests of normality and adjustment for multiple comparisons |
| ☐ | ☒ | A full description of the statistical parameters including central tendency (e.g. means) or other basic estimates (e.g. regression coefficient) AND variation (e.g. standard deviation) or associated estimates of uncertainty (e.g. confidence intervals) |
| ☐ | ☒ | For null hypothesis testing, the test statistic (e.g. *F*, *t*, *r*) with confidence intervals, effect sizes, degrees of freedom and *P* value noted<br>*Give P values as exact values whenever suitable.* |
| ☒ | ☐ | For Bayesian analysis, information on the choice of priors and Markov chain Monte Carlo settings |
| ☒ | ☐ | For hierarchical and complex designs, identification of the appropriate level for tests and full reporting of outcomes |
| ☒ | ☐ | Estimates of effect sizes (e.g. Cohen's *d*, Pearson's *r*), indicating how they were calculated |

*Our web collection on statistics for biologists contains articles on many of the points above.*

## Software and code

Policy information about availability of computer code

| | |
|---|---|
| Data collection | Single cell RNA-seq libraries for the longitudinal dataset (wt/hog1 control or 0.4M NaCl), qas well as the transcription focused transcriptional profilig were performed using 10X genomics (Single Cell 3' v3). Cells were harvested and fixed with methanol. For each condition a single 10X lane was used. Libraries for each dataset were performed in parellel and pooled together for sequencing using paired end sequencing (Illuimna). Cell fixation and library preparation were performed according to manufacturer's protocol. The resulting FASTQ files were sued as an input for pre-processing using cell ranger pipeline(v4.0.0). |
| Data analysis | For the data analysis. The CellRanger software (10X genomics) was used using default parameters. The output folders for all samples (transcriptome libraries) were used as an input to generate Seurat objects and R Studio and published R packages we used. The raw data has been subitted to AGEO the code to reprodice the figures and the processed Seurat objects been also uploaded to Zenodo Direct links or accession number are provided within the manuscript. |

For manuscripts utilizing custom algorithms or software that are central to the research but not yet described in published literature, software must be made available to editors and reviewers. We strongly encourage code deposition in a community repository (e.g. GitHub). See the Nature Portfolio guidelines for submitting code & software for further information.

## Data

Policy information about availability of data

All manuscripts must include a data availability statement. This statement should provide the following information, where applicable:

- Accession codes, unique identifiers, or web links for publicly available datasets
- A description of any restrictions on data availability
- For clinical datasets or third party data, please ensure that the statement adheres to our policy

The raw sequencing data and pre-processed data for the longitudinal scRNA-seq profiling and the transcription targeted deletion profiling Gene Expression Omnibus GSE274661 [https://www.ncbi.nlm.nih.gov/geo/query/acc.cgi?acc=GSE274661]. Source data are provided as a Source Data file.

Code availability
All the code used in this study is available through Zenodo: 10.5281/zenodo.13731922 [https://doi.org/10.5281/zenodo.13731922]

## Research involving human participants, their data, or biological material

Policy information about studies with human participants or human data. See also policy information about sex, gender (identity/presentation), and sexual orientation and race, ethnicity and racism.

| | |
|---|---|
| Reporting on sex and gender | does not apply |
| Reporting on race, ethnicity, or other socially relevant groupings | does not apply |
| Population characteristics | does not apply |
| Recruitment | does not apply |
| Ethics oversight | does not apply |

Note that full information on the approval of the study protocol must also be provided in the manuscript.

# Field-specific reporting

Please select the one below that is the best fit for your research. If you are not sure, read the appropriate sections before making your selection.

☒ Life sciences ☐ Behavioural & social sciences ☐ Ecological, evolutionary & environmental sciences

For a reference copy of the document with all sections, see nature.com/documents/nr-reporting-summary-flat.pdf

# Life sciences study design

All studies must disclose on these points even when the disclosure is negative.

| | |
|---|---|
| Sample size | No statistical methods were used to calculate sample size. |
| Data exclusions | For scRNA-seq, cells that did not meet the QC were removed. In addition cells that passed the QC but the genotype barcode could not assign to a genotype or were assigned to more than one genotype were removed. When specified only genotypes with at least 6 cells were considered to avoid noise from low abundant genotypes. |
| Replication | Experimental validations of the scRNA-seq dataset derive from three independent biological replicates. For the scRNA-seq dataset one replicate per condition. |
| Randomization | FACS analysis the entire single population was used to define either population abundance or gene expression. For the rest of the experiments not mentioned here randomization was not important. |
| Blinding | Generation of scRNA-seq libraries after cell partition including sequencing and data preprocessing (alignment and genotype calling) were done blindly. |

# Reporting for specific materials, systems and methods

We require information from authors about some types of materials, experimental systems and methods used in many studies. Here, indicate whether each material, system or method listed is relevant to your study. If you are not sure if a list item applies to your research, read the appropriate section before selecting a response.

## Materials & experimental systems

| n/a | Involved in the study |
|---|---|
| ☒ | ☐ Antibodies |
| ☒ | ☐ Eukaryotic cell lines |
| ☒ | ☐ Palaeontology and archaeology |
| ☐ | ☒ Animals and other organisms |
| ☒ | ☐ Clinical data |
| ☒ | ☐ Dual use research of concern |
| ☒ | ☐ Plants |

## Methods

| n/a | Involved in the study |
|---|---|
| ☒ | ☐ ChIP-seq |
| ☐ | ☒ Flow cytometry |
| ☒ | ☐ MRI-based neuroimaging |

# Animals and other research organisms

Policy information about studies involving animals; ARRIVE guidelines recommended for reporting animal research, and Sex and Gender in Research

| | |
|---|---|
| Laboratory animals | does not apply |
| Wild animals | does not apply |
| Reporting on sex | does not apply |
| Field-collected samples | does not apply |
| Ethics oversight | does not apply |

Note that full information on the approval of the study protocol must also be provided in the manuscript.

# Plants

| | |
|---|---|
| Seed stocks | does not apply |
| Novel plant genotypes | does not apply |
| Authentication | does not apply |

# Flow Cytometry

## Plots

Confirm that:

☒ The axis labels state the marker and fluorochrome used (e.g. CD4-FITC).

☒ The axis scales are clearly visible. Include numbers along axes only for bottom left plot of group (a 'group' is an analysis of identical markers).

☐ All plots are contour plots with outliers or pseudocolor plots.

☒ A numerical value for number of cells or percentage (with statistics) is provided.

## Methodology

| | |
|---|---|
| Sample preparation | Samples analyzed by Flow cytometry were analyzed directly from the growth media. For expression of reporters cells were analyzed in expoentnial phase. |
| Instrument | For analysis: Cytek® Aurora (4-laser and 64 Fluorescence Emission Detection Channels) |
| Software | Cytometry data were analyzed using FlowJo™ Software (BD Life Sciences). |

| | |
|---|---|
| Cell population abundance | For reporter analysis a total of 10,000 cells were used to determine expression distribution.<br>For competition assays at time 0 a total of 1000 cells were used to determine initial cell ratios.. |
| Gating strategy | The gating strategy follows standard procedures where cell population is selected through gating cell size and complexity (FSC and SSC) using the FlowJo. The degree of expression reported is always represented from this gate. |

☒ Tick this box to confirm that a figure exemplifying the gating strategy is provided in the Supplementary Information.
